# Players are positive regarding injury prevention exercise programmes, but coaches need ongoing support: a survey-based evaluation using the Health Action Process Approach model across one season in amateur and youth football

**DOI:** 10.1136/bmjsem-2024-002009

**Published:** 2024-06-24

**Authors:** Hanna Lindblom, Ida Åkerlund, Markus Waldén, Sofi Sonesson, Martin Hägglund

**Affiliations:** 1 Unit of Physiotherapy, Department of Health, Medicine and Caring Sciences, Linköping University, Linköping, Sweden; 2 Sport Without Injury ProgrammE (SWIPE), Department of Health, Medicine and Caring Sciences, Linköping University, Linköping, Sweden; 3 Capio Ortho Center Skåne, Malmö, Sweden

**Keywords:** Exercise, Behaviour, Prevention, Football, Sporting injuries

## Abstract

**Objectives:**

Implementation of injury prevention exercise programmes (IPEPs) in sports is challenging, and behaviour change among players and coaches is essential for success. The aim was to describe players’ and coaches’ motivation and coaches’ goal pursuit when using IPEPs in amateur and youth football across a season. A secondary aim was to describe players’ motivation to engage in IPEP use in relation to presence or absence of injury.

**Methods:**

The study was based on questionnaires to amateur and youth, male and female football players and coaches at baseline, mid-season and post-season in a three-armed randomised trial in 2020 in Sweden. Questionnaires were based on the Health Action Process Approach (HAPA) model with questions about the motivational phase when intention for change is created (players and coaches) and a goal-pursuit phase when intention is translated into action (coaches).

**Results:**

In total, 455 players (126 male), mean age 20.1 years (SD±5.8, range 14–46) and 59 (52 male) coaches took part. Players generally gave positive answers in the HAPA motivational phase (Likert 6–7 on a 1–7 Likert scale). Differences in ratings between injured and uninjured players were minor. Coaches had positive or neutral ratings (Likert 4–6) in the motivational and goal-pursuit phases. Ratings deteriorated across the season, with less positive responses from 40% of players and 38-46% of coaches post-season.

**Conclusion:**

Positive ratings in the HAPA motivational phase indicated fertile ground for IPEP use. Neutral ratings by coaches and deterioration across the season in players and coaches suggest a need for ongoing support for IPEP use.

**Trial registration number:**

NCT04272047.

**WHAT IS ALREADY KNOWN ON THIS TOPIC**

- Implementation of injury prevention exercise programmes (IPEPs) is dependent on behaviour change in players and coaches.
- Coaches tend to modify IPEP content or dosage, and players are reported to have low motivation for injury prevention, which together challenges the successful prevention of injuries in sports.

**WHAT THIS STUDY ADDS**

- The study covered both the motivational (players and coaches) and the goal-pursuit (coaches) phases of the Health Action Process Approach model across one football season and in relation to three different IPEPs.
- Players showed high motivation for IPEP use, and differences were small between ratings from players using different IPEPs, between males and females, and between injured and uninjured players. Findings were not in agreement with previous studies describing low player motivation as a barrier to IPEP use.
- Coaches were neutral or positive regarding their motivation for IPEP use, and their goal pursuit to start and maintain use of IPEPs over time. Specifically, they rated neutrally on their belief in their ability to use an IPEP (action self-efficacy), their plans for instructing players, and their plans to work around barriers for continued IPEP use, suggesting need for continuous support.

**HOW THIS STUDY MIGHT AFFECT RESEARCH, PRACTICE OR POLICY**

- The finding of motivated players was encouraging, but in the future, other research designs, such as qualitative studies and studies outside the controlled context of a randomised trial, may enable a deeper understanding of the player perspective of injury prevention.
- Strategies including workshops at the beginning of the season introducing injury prevention exercise programmes (IPEPs) may be supplemented by ongoing strategies throughout the season to support coaches in formalising plans for IPEP use over time and to support coaches’ self-efficacy beliefs in relation to injury prevention. These ongoing initiatives may also potentially prevent the deterioration in motivation and goal pursuit shown across the season among both players and coaches.

## Introduction

Injury prevention exercise programmes (IPEPs) have been proven effective in reducing lower limb injury rates in various team sports.^1–6^ Due to programme modification^7–10^ and low player exercise fidelity,^11–14^ IPEPs often do not reach their full potential and optimal real-world implementation is a challenge.^15^ Players and coaches are important stakeholders when implementing and using IPEPs.^16^ Whereas players are the intended end-users, coaches usually have the mandate to determine whether, when and how IPEPs are used, and they often lead the preventive training for the whole team. Previous studies have reported low player motivation as a barrier that potentially affects the use of the IPEPs.^8 17–19^ Considering the gap between efficacy and effectiveness in injury prevention,^20 21^ there is a need for a better understanding of factors that drive behaviour change, in the adoption and maintained use of IPEPs. The Health Action Process Approach (HAPA) is a two-phase behaviour change model, with a motivational phase when intention for change is created and a volitional, goal-pursuit phase where this intention is translated into action.^22^ Risk perceptions, outcome expectancies, action self-efficacy and intention are key constructs in the motivational phase, whereas action and coping planning, maintenance and recovery self-efficacy constitute the goal-pursuit phase.^23^ The HAPA model has been applied previously in amateur and youth sport contexts in football, rugby union and floorball.^10 18 24–26^ By identifying a need to strengthen specific constructs, targeted measures to improve adoption and maintained use of IPEPs may be developed. Hence, the aim was to describe players’ and coaches’ motivation and coaches’ goal pursuit when using IPEPs in amateur and youth football across a season. A secondary aim was to describe players’ motivation to engage in IPEP use in relation to presence or absence of injury.

## Methods

This study was based on questionnaire data collected at baseline, mid-season and post-season from players and coaches in a three-armed randomised trial that was conducted in Sweden in 2020.^6^ Teams were randomised to a further developed version of the IPEP *Knee Control*, the *extended Knee Control*,^6^ or a short single-exercise adductor strength programme based on the *Adductor strengthening programme*.^27^ Teams that already used an IPEP on regular basis at study start were not randomised but were allocated to a comparison group that continued with their usual training throughout the season and did not receive any intervention as part of the study.

In this subanalysis, data are presented from players and coaches who responded to the baseline questionnaire and the mid-season and/or post-season questionnaires. Baseline questionnaires were distributed before the competitive season in April, mid-season questionnaires in June before the summer break and post-season questionnaires in October/November, depending on when the team’s season ended. The total season ranged in length from 27 to 29 weeks. Since the study was conducted during the COVID-19 pandemic, the preseason was extended, and the competitive season was postponed. However, football training was never cancelled in Sweden and injury prevention training continued throughout the season. The study was checked against the Strengthening the Reporting of Observational Studies in Epidemiology checklist.^28^

### Participants

Players ≥14 years of age and coaches in teams who participated in a male or female adolescent or adult league 2020 series in one football district and who had at least two scheduled training sessions per week were eligible. Teams in the randomised arms had not engaged in regular prevention training during the previous year, while teams in the non-randomised arm (comparison group) had used an IPEP regularly at least once per week during the previous year and planned to do so again in the 2020 season. All coaches for these teams who we had contact information for were eligible. In total, 17 teams were randomised to *extended Knee Control*, 12 teams were randomised to the adductor programme and 17 teams were allocated to the comparison group.

### Interventions

Only information relevant to this substudy is presented here, a detailed description of the interventions has previously been published.^6^ Teams in the randomised groups were offered workshops or site visits where the respective intervention, *extended Knee Control* or the adductor programme, was introduced and exercises were practised (table 1). Afterwards, coaches were expected to lead the preventive training in their team. Both randomised groups also received printed and digital programme materials. Teams were instructed to begin training immediately after workshops or site visits and to carry on with preventive training throughout the season.

**Table 1:** Description of the interventions in the randomised groups

	*Extended Knee Control*	Adductor programme
Exercises	Running warm-up (5 min) 6 main strengthening and neuromuscular control exercises (10 variations for each) (10–15 min): One-legged knee squat Hamstring strengthening Two-legged knee squat Core strengthening Lunge Jump/landing technique	One exercise out of: Copenhagen adduction, long lever Copenhagen adduction, short lever Side-lying adduction Adductor squeeze (ball between knees, bent legs) Adductor squeeze (ball between feet, straight legs)
Frequency	Every training session throughout the season Running warm-up before matches	2–3 times/week preseason 1 time/week competitive season
Dosage	30–60 s per exercise×2 sets	3–5 to 12–15 repetitions (Copenhagen adduction and Side-lying adduction) or 10 s maximal isometric contractions×5 repetitions (Adductor squeeze) 1 set
Programme material	Printed programme folder Digital folder Films and instructions on website	Printed programme folder Digital folder Films and instructions on website

### Questionnaires

Web-based questionnaires were distributed via a link that was sent by email and/or short message service to players and coaches and complemented by two reminders to non-responders. The questionnaires were bespoke but based on a previous questionnaire applying the HAPA model.^18^ All questions were responded on a 1–7 Likert scale, where 1 represented the least and 7 the most favourable option. Baseline questionnaires were distributed to all coaches after intervention group coaches had taken part in workshops or site visits and to players at the same time.

Players and coaches responded to questions related to the motivational phase in HAPA at all three time points (table 2). Coaches also responded to questions relating to the goal-pursuit phase. Most questions in the goal-pursuit phase were asked at mid-season and post-season. Players received questions about the motivational phase only since motivation for IPEP use may impact their adherence and fidelity to prevention programme use but did not receive questions related to the goal-pursuit phase since the decision to use an IPEP usually lies with the coach. In the interest of brevity and to reduce response burden, we strived to limit the number of questions to players and also adapted questions to better suit each group, players and coaches, based on their specific roles and responsibilities related to IPEP use. Therefore, questions are similar, but not identical, for players and coaches.

**Table 2:** Distribution of questions in relation to the two Health Action Process Approach (HAPA) phases

	Players	Coaches
Baseline	4 questions	8 questions
Motivational phase	Injury risk perceptions 1 question Outcome expectancies 2 questions Intention 1 question	Injury risk perceptions 2 questions Outcome expectancies 2 questions Intention 1 question Action self-efficacy 2 questions
Goal-pursuit phase	n/a	Action planning 1 question
Mid-season	8 questions	11 questions
Motivational phase	Same 4 as baseline + action self-efficacy 3 questions + attitude towards continued IPEP training 1 question	Same 7 questions as baseline
Goal-pursuit phase	n/a	Same question as baseline Maintenance self-efficacy 1 question Coping planning 1 question Recovery self-efficacy 1 question
Post-season	8 questions, same as mid-season but slightly different wording focusing on next season instead of present season	11 questions, same as mid-season but slightly different wording focusing on next season instead of present season

Baseline questionnaires were distributed in April, approximately 2 months before the competitive season, mid-season questionnaires were distributed in June before the summer break and post-season questionnaires were distributed in October/November.

IPEP, injury prevention exercise programme; n/a, not applicable.

In addition to the HAPA questions, players were asked at baseline whether they had any ongoing or previous injuries (anytime previously in their career) in the hip/groin, hamstring, knee or ankle. These four injury locations were the primary prevention targets in the main randomised trial. During the season, players reported the occurrence of injury in a weekly questionnaire based on the Oslo Sports Trauma Research Centre (OSTRC) questionnaire (OSTRC-O2).^29^ For this substudy, players who reported any physical complaint in any body location during the season were treated as ‘injured’.

### Statistical analysis

No sample size calculation was made for this subanalysis, only for the main study.^6^ Results are presented descriptively with medians and IQR for each question as well as aggregated per HAPA construct. Aggregated construct scores are presented in the main manuscript, whereas raw scores for each question are presented in the online supplemental file. The distribution of responses across the 1–7 Likert scale is presented in figures. Likert responses 1–2 are considered negative, 3–5 neutral and 6–7 positive. Responses at baseline are also presented separately based on whether the players entered the study with ongoing or previous injury or not. Post-season ratings of players who reported an injury during the season are contrasted with ratings of players who did not report an injury. When presenting ratings stratified by sex (player) or intervention group (players and coaches), we use the post-season ratings since respondents had had most time to experience the interventions during post-season. We also applied Paretian principles in an analysis and compared ratings within the same HAPA phase across the season for each individual relating to whether ratings improved, were unchanged, deteriorated or were indeterminable (where deteriorations were seen in some constructs and improvements in other constructs). This has previously been described for analyses of the EuroQol 5-Dimension for health-related quality of life.^30^ In our analysis, Likert responses were combined into three categories: Likert responses 1–2, 3–5 and 6–7, and a change was only considered when an individual’s rating differed from one category to another between time points. The analysis only includes participants with data from both analysed time points (baseline and post-season or mid-season and post-season). No missing data were imputed.

10.1136/bmjsem-2024-002009.supp1 Supplementary data

### Patient and public involvement

*Extended Knee Control* programme development was informed by a qualitative study with coaches^8^ and pilot-tested with players and coaches,^31^ but players or coaches did not take part in the planning or conduct of the study.

## Results

In total, 455 (126 male) players (mean age 20.1 years (SD 5.8, range 14–46)) responded to the baseline questionnaire, corresponding to 91% of the players who took part in the main study.^6^ In total, 59 (52 male) coaches (mean age 43.9 years (SD 9.0)) responded to the baseline questionnaire representing 40 teams (87% of participating teams), and an additional 2 teams, where coaches took part in workshops but ended their participation afterwards, whose players never entered the study. 15 coached male youth or senior teams, 44 coached female youth or senior teams.

### Ratings in the motivational phase among players

**Figure 1:**
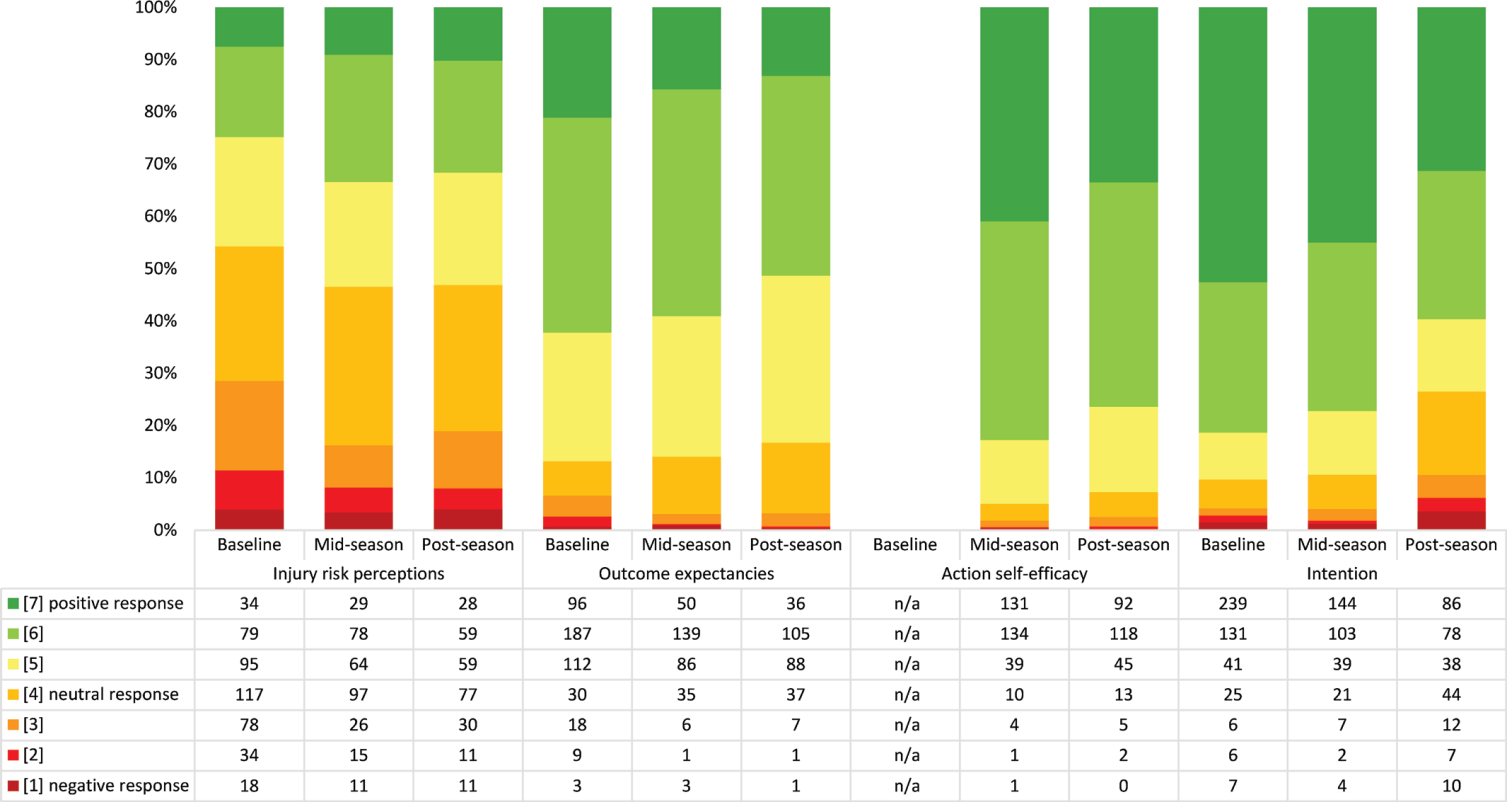
Distribution of player responses in the constructs in the HAPA model motivational phase across one season. n=455 at baseline, n=320 at mid-season and n=275 at post-season. Action self-efficacy was only rated at mid-season and post-season. For constructs where players responded to more than one question, the averaged aggregated responses are shown in the figure. HAPA, Health Action Process Approach; n/a, not applicable

Ratings in the motivational phase were either neutral (injury risk perceptions) or generally positive (outcome expectancies, action self-efficacy and intention) among players at both baseline and across the season (figure 1, online supplemental table 1). Differences in ratings in players in different intervention groups were minor (±1 point on the Likert scale) (online supplemental table 2, online supplemental figures 1–3). At post-season, male players seemed slightly more negative than female players in all four constructs (online supplemental figure 4).

### Changes in player ratings across one season

In the analysis applying Paretian principles and evaluating changes from baseline to post-season, 65 players (24%) had unchanged ratings in the motivational phase, 109 players (40%) deteriorated (rated a less positive response), 58 players (21%) improved (had more positive ratings in post-season) and 43 players (16%) were indeterminable (ie, had both improved and deteriorated ratings in different constructs). When scrutinising each separate construct in the motivational phase, most ratings in injury risk perceptions (58%), outcome expectancies (63%) and intention (61%) were unchanged from baseline to post-season (online supplemental figures 5 and 6).

### Player motivation in relation to injury status

**Figure 2:**
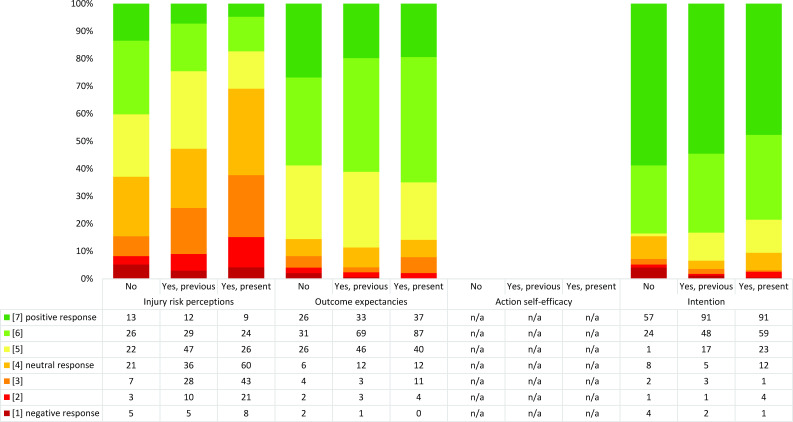
Baseline ratings of constructs in the HAPA model motivational phase separated for players with previous or present injury or without injury at baseline. Players with both previous and present injury are represented under present injury. n=455. Action self-efficacy was only rated at mid-season and post-season. For outcome expectancies, where players responded to more than one question, the averaged aggregated responses are shown in the figure. HAPA, Health Action Process Approach; n/a, not applicable.

Baseline ratings from players who entered the study with or without injury are presented in figure 2. Notably, injury risk perceptions seem to differ, with more injured players rating negative responses, that is, they believed to a higher extent that they would incur an injury during the season. Differences in outcome expectancies or intention to take part in injury prevention training were negligible between injured/non-injured players. Differences in post-season ratings between players who incurred an injury during the season or not were also negligible (online supplemental figures 7 and 8).

### Ratings in the motivational phase among coaches

**Figure 3:**
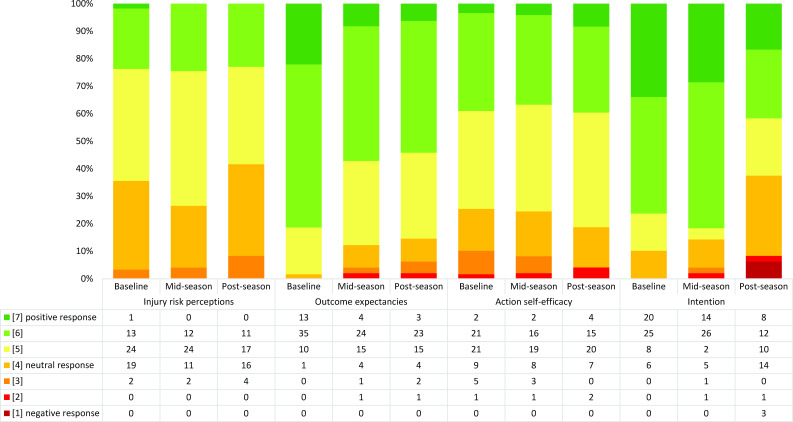
Distribution of coach responses in the constructs in the HAPA model motivational phase across one season. n=59 at baseline, n=49 at mid-season and n=48 at post-season. For constructs where coaches responded to more than one question, the averaged aggregated responses are shown in the figure. HAPA, Health Action Process Approach.

Coaches’ ratings in the motivational phase were neutral (injury risk perceptions, action self-efficacy) or positive (outcome expectancies, intention) at baseline (figure 3, online supplemental table 3). Slightly more positive ratings were seen in the *extended Knee Control* group, and there were fewer positive ratings in the other two groups at post-season regarding outcome expectancies and action self-efficacy (online supplemental figure 9).

### Ratings in the goal-pursuit phase among coaches

**Figure 4:**
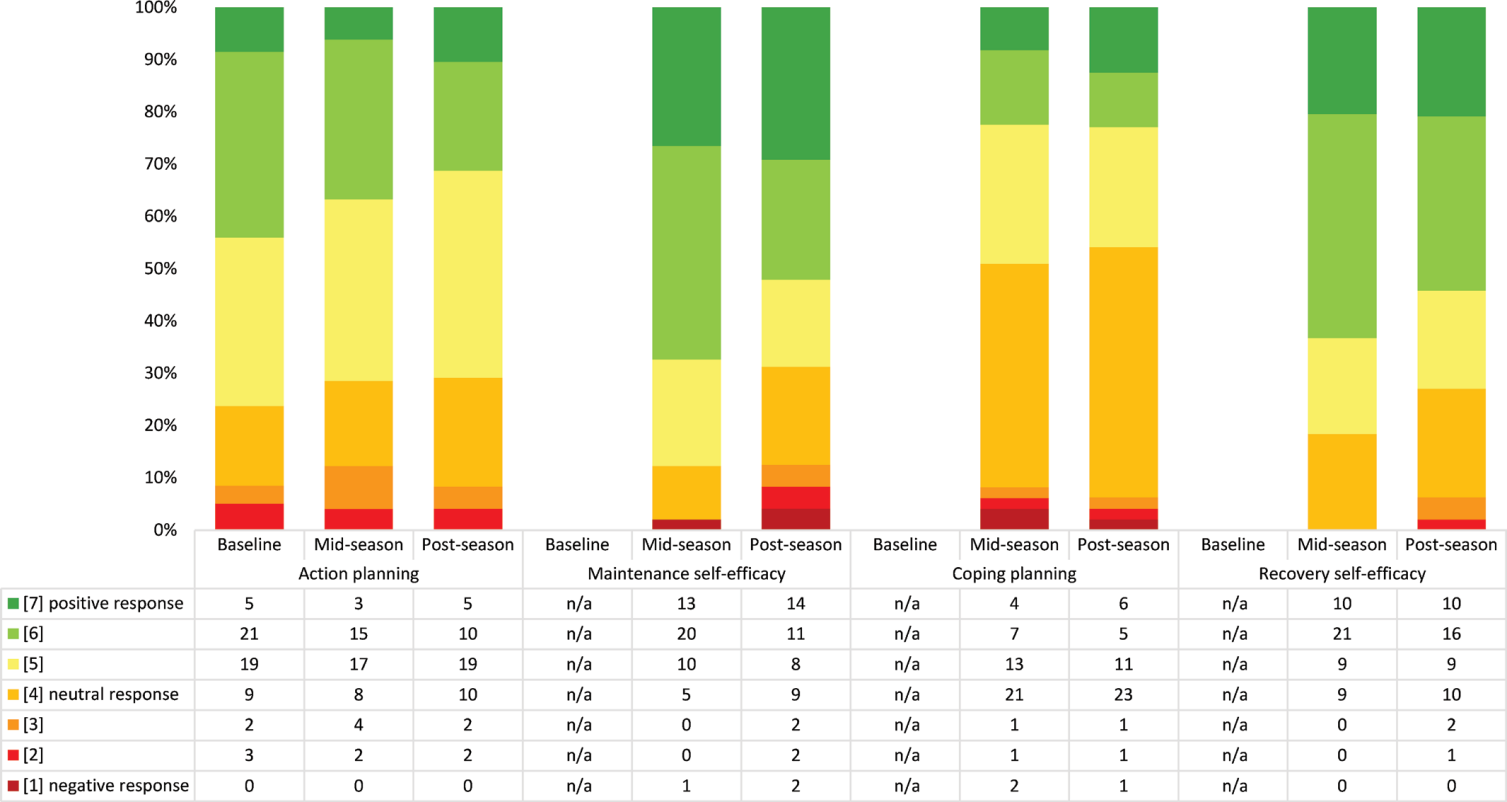
Distribution of coach responses in the constructs in the HAPA model goal-pursuit phase across one season. n=59 at baseline, n=49 at mid-season and n=48 at post-season. Maintenance self-efficacy, coping planning and recovery self-efficacy were only rated at mid-season and post-season. For constructs where coaches responded to more than one question, the averaged aggregated responses are shown in the figure. HAPA, Health Action Process Approach; n/a, not applicable.

In the goal-pursuit phase, coaches rated positively regarding maintenance and recovery self-efficacy but were neutral about action and coping planning (figure 4, online supplemental table 3). Slight differences were seen during post-season, with more positive responses in the *extended Knee Control* group and more negative responses in the comparison group (the adductor group being somewhere in between) (online supplemental figure 10).

### Change in ratings in the motivational and goal-pursuit phases among coaches across one season

The analysis applying Paretian principles for the motivational phase from baseline to postseason showed that 17 coaches (35%) had unchanged ratings, 22 (46%) deteriorated, 5 (10%) improved and 4 (8%) were indeterminable (online supplemental figure 11). The analysis applying Paretian principles for the goal-pursuit phase showed that 11 coaches (24%) had unchanged ratings, 17 (38%) deteriorated, 12 (27%) improved and 5 (11%) were indeterminable (online supplemental figure 12).

## Discussion

In this study on behavioural support for the use of IPEPs, we found motivation for IPEP use among both players and coaches, and positive responses in the goal-pursuit phase among coaches, with neutral or positive ratings in each of the constructs, suggesting fertile ground for IPEP use. Ratings were similar irrespective of intervention group among players, but coaches in *extended Knee Control* seemed more positive than coaches in the other two groups. Overall, ratings deteriorated slightly across the season, as depicted in the analysis applying Paretian principles which showed that 38%–46% of players and coaches had lower—that is, less positive—ratings in post-season compared with baseline (motivational phase) or mid-season (goal-pursuit phase). Differences between players who had previously incurred or incurred a new injury during the season in contrast to players without injury were minor.

Player ratings were surprisingly positive, considering previous studies describing low motivation for IPEP use among players.^8 17–19^ However, the present study was carried through in the context of a randomised trial, where possibly only the most motivated coaches and teams choose to take part, and where the amount of support for IPEP use differs compared with studies in a real-world context. Based on the differences seen in the present study, male players may be slightly more negative toward IPEP use than female players. Considering the focus on knee injuries in early studies on IPEPs, players and coaches may believe that the programmes are more for women. Hence, it would be of value to clarify to players and coaches that the programmes target injuries in the lower extremity overall in both males and females. The high action self-efficacy—with players describing high confidence in their ability to do the preventive exercises, their effort in doing so and their ability to listen to the coach’s instructions—was positive. However, considering the low exercise fidelity when using the *Knee Control* exercises in football,^11^ either the players are unrealistic, or unable to estimate their own ability in a reliable way; for example, due to insufficient information from coaches about the performance of the exercises. In summary, there seems to be more to this than these current questionnaires can capture and further formalised development of the questionnaires, as well as establishment of their construct validity, responsiveness for change and test–retest reliability is one way forward to learn more about the player’s views. Another important way forward is to conduct qualitative studies to extend our understanding of the player’s perspective.

In the motivational phase, coaches rated positive regarding outcome expectancies and intention, suggesting that they are aware of the benefits of injury prevention and intend to use IPEPs. Risk perceptions received lower ratings, but have been shown previously to not be as significant as outcome expectancies and action self-efficacy when forming intention for IPEP use.^18 25^ Coaches were neutral regarding action self-efficacy, suggesting that this may be an area for further improvement. This is in line with previous studies describing low self-efficacy among coaches and that they are unsure whether they are using the programme in the right way.^8^ Positive effects on self-efficacy have been shown after taking part in IPEP workshops,^10 26^ suggesting there is potential to improve self-efficacy. In the goal-pursuit phase, coaches rated maintenance and recovery self-efficacy high; however, they had lower ratings regarding action and coping planning. This does not align with the previous study in rugby union, where strong correlations were shown between maintenance self-efficacy and action planning.^25^ This discrepancy is possibly related to the fact that the present study only covered each construct in the goal-pursuit phase with one question, making this measurement less precise and, again, suggesting a need for further development of the questionnaires. During the study, we mainly focused on supporting coaches to adopt IPEPs by offering workshops at the study start and did not specifically target maintained use of IPEPs. This may also be a reason for the deteriorations in ratings that we noticed in the analysis applying Paretian principles from baseline or mid-season to post-season. The deteriorations seen among almost half of the players are more difficult to explain but clearly indicate that coaches and players need ongoing support to ensure maintained use, and we should not assume that they will continue to use the programme effortlessly. The results, with relatively minor changes in median ratings over time and between subgroups, tally with a study in school physical education classes^32^ but contradict the findings from Barden *et al*,^25^ who showed that taking part in workshops improved action self-efficacy and intention to use an IPEP among rugby coaches after the season, compared with before. In line with this study, we also included activities aiming to support high coach self-efficacy in delivering the IPEPs, however, intention and planning activities may have been given more attention in the study by Barden *et al*.^25^ In the present study, coaches in the comparison group who did not receive any intervention or specific support were the ones who showed negative ratings in post-season. This emphasises the need for ongoing support also among those who regularly use IPEPs. To further elucidate how this support may be structured, qualitative studies examining the experiences of and need for support for IPEP use in more depth would be valuable.

Even though median values were high, we noticed a high spread in the responses from both players and coaches; that is, there were both positive and negative responses. This suggests that it may be futile to develop standardised support that fits all coaches and teams, and that a better way may be to develop intervention material made up of different parts that can be added or removed as needed, that is, a smorgasbord. Argumentation away from standardised intervention approaches has also been published elsewhere.^33^ Considering that the results of the present study are comparable with previous studies in the realworld outside the controlled context of a randomised trial,^9^ regarding the 11*+* 18 and in youth floorball,^26^ the results may probably be generalisable to other team sports and IPEPs.

### Limitations

The strengths of this study included the good representation of the player perspective, totalling 455 players at baseline. Another strength was the inclusion of both player and coach perspectives in the same study. Even though the questionnaires were not formally validated, they were theoretically based on a behaviour change model, the HAPA model, which is a strength. Similar questions and Likert scale ratings have also been used in previous studies.^9 10 18 24–26^

The study is limited by the fact that we do not know whether statistical differences in the Likert scores between groups or over time can be regarded as clinically meaningful. For this reason, we chose not to make statistical comparisons between groups or across the season but rather present results descriptively. In circumstances when youth and senior players had the same coach and had football training together, we lack information about which league each player played in and, therefore, only present results mixed for youth and senior players. Another limitation was the relatively small sample of coaches which does not allow for group comparisons. Considering the questionnaire, there are only a few questions per construct, and not all constructs may be properly covered by this small number of questions. To really be able to capture separate constructs, we may need to use other questionnaires, such as a self-efficacy scale, to delve more deeply into this construct. However, the present questions may still indicate target areas for future implementation strategies. Last, but not least, this study was accomplished during the first year of the COVID-19 pandemic. Even though football training continued throughout the pandemic in Sweden, players and coaches were obviously affected by the restrictions in the community and the spread of the disease, and their ratings may have been affected by this uncertainty. When comparing ratings to a 2021 study performed in the same geographical district,^9^ we notice ratings in the same HAPA constructs that are about 1 point lower on the Likert scale in the present study.

## Clinical implications

Players and coaches had positive ratings in the HAPA motivational phase, indicating fertile ground for IPEP use. Neutral ratings by coaches on action self-efficacy, action planning and coping planning as well as a deterioration in scores across the season in both players and coaches, suggest that they should be offered ongoing support for IPEP use during the season in addition to initial workshops at the beginning of the season.

## Data Availability

All data relevant to the study are included in the article or uploaded as online supplemental information. All data generated or analysed during this study are included in this published article (and its online supplemental information files).
