# Supplementary material for: Players are positive regarding injury prevention exercise programmes, but coaches need ongoing support: a survey-based evaluation using the Health Action Process Approach model across one season in amateur and youth football
Source: BMJ Open Sport Exerc Med. 2024 Jun 24;10(2):e002009. doi: 10.1136/bmjsem-2024-002009 (PMC11202753; doi:10.1136/bmjsem-2024-002009)
Supplement: Supplementary data [file bmjsem-2024-002009supp001.pdf]

Supplement

Supplementary Figure 1. Presentation of player responses in the constructs in the HAPA model motivational phase across one season in the *extended Knee Control* group.

Supplementary Figure 2. Presentation of player responses in the constructs in the HAPA model motivational phase across one season in the Adductor group.

Supplementary Figure 3. Presentation of player responses in the constructs in the HAPA model motivational phase across one season in the Comparison group.

Supplementary Figure 4. Presentation of player responses in the constructs in the HAPA model motivational phase at post-season, stratified by player sex.

Supplementary Figure 5. Results from the analysis applying Paretian principles for the player ratings in the HAPA model motivational phase from baseline to post-season.

Supplementary Figure 6. Change in the ratings in injury risk perceptions, outcome expectancies, and intention for players in the respective groups from baseline to post-season.

Supplementary Figure 7. Ratings in the constructs in the HAPA model across one season for players who did not incur an injury during the season.

Supplementary Figure 8. Ratings in the constructs in the HAPA model across one season for players who incurred an injury during the season.

Supplementary Figure 9. Presentation of coach responses in the constructs in the HAPA model motivational phase at post-season separated by intervention group.

Supplementary Figure 10. Presentation of coach responses in the constructs in the HAPA model goal-pursuit phase, separated by intervention group.

Supplementary Figure 11. Results from the analysis applying Paretian principles for the coach ratings in the HAPA model motivational phase from baseline to post-season.

Supplementary Figure 12. Results from the analysis applying Paretian principles for the coach ratings in the HAPA model goal-pursuit phase from mid-season to post-season.

Supplementary Table 1. Player ratings in the HAPA model motivational phase across one season.

Supplementary Table 2. Player ratings in the HAPA model motivational phase separated by intervention group.

Supplementary Table 3. Coach ratings in the HAPA model motivational and goal-pursuit phases.

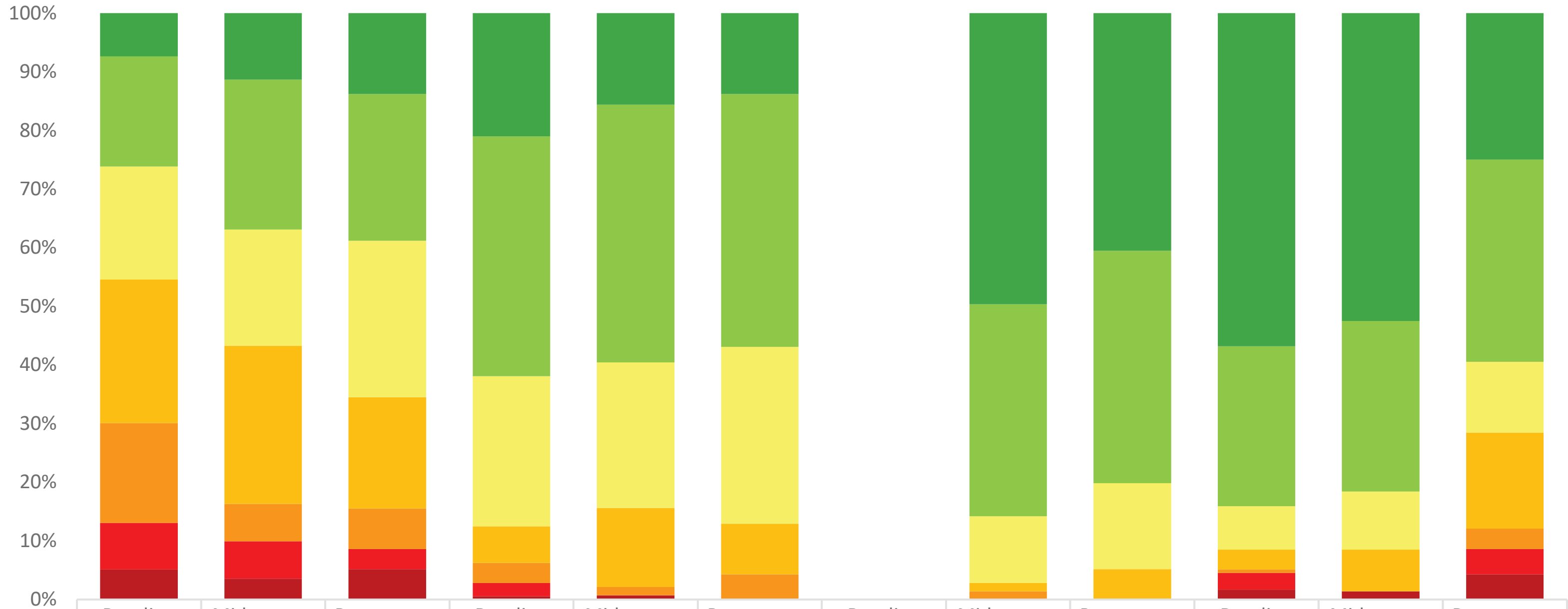

	Injury risk perceptions			Outcome expectancies			Action self-efficacy			Intention		
	Baseline	Mid-season	Post-season	Baseline	Mid-season	Post-season	Baseline	Mid-season	Post-season	Baseline	Mid-season	Post-season
[7] positive response	13	16	16	37	22	16	n/a	70	47	100	74	29
[6]	33	36	29	72	62	50	n/a	51	46	48	41	40
[5]	34	28	31	45	35	35	n/a	16	17	13	14	14
[4] neutral response	43	38	22	11	19	10	n/a	2	6	6	10	19
[3]	30	9	8	6	2	5	n/a	2	0	1	0	4
[2]	14	9	4	4	0	0	n/a	0	0	5	0	5
[1] negative response	9	5	6	1	1	0	n/a	0	0	3	2	5

Supplementary Figure 1. Presentation of player responses in the constructs in the HAPA model motivational phase across one season in the extended Knee Control group.

n = 176 at baseline, n = 141 at mid-season and n =116 at post-reason. Note: Action self-efficacy was only rated at mid- and post-season. For constructs where players responded to more than one question, the averaged aggregated responses are shown in the figure. Abbreviations: HAPA — Health Action Process Approach, n/a — not applicable.

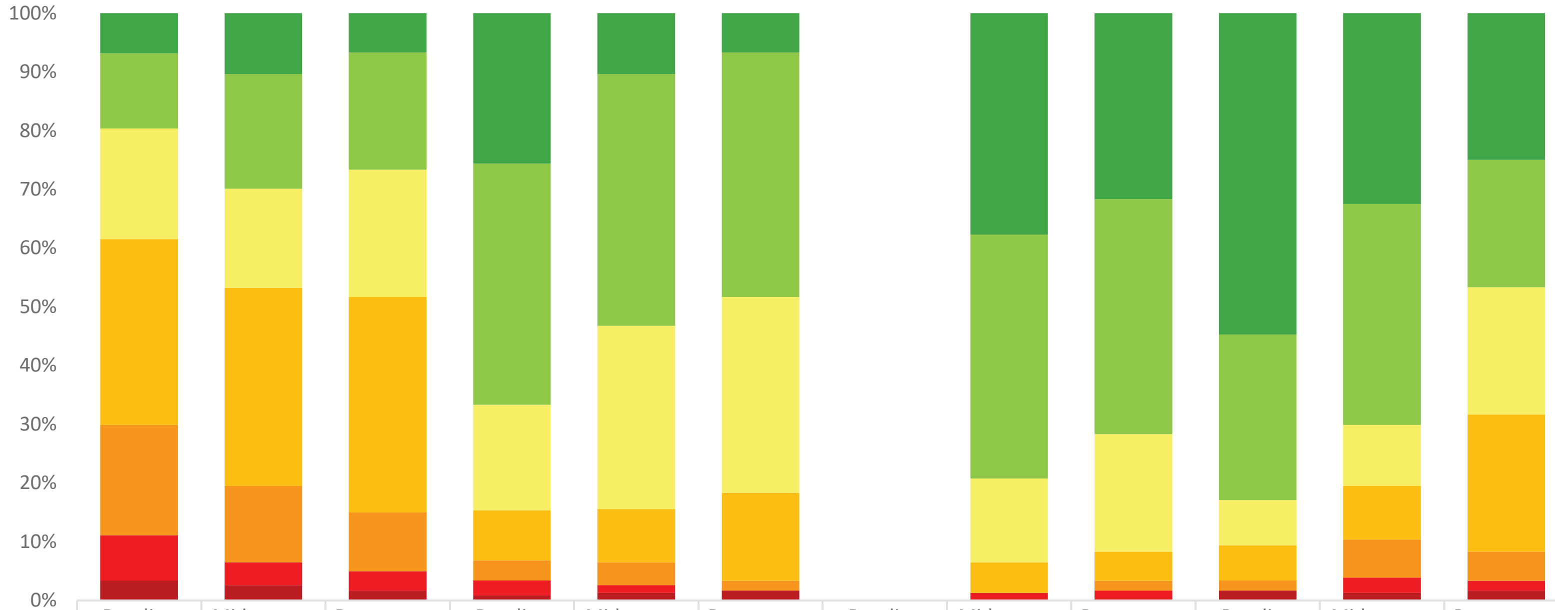

	Injury risk perceptions			Outcome expectancies			Action self-efficacy			Intention		
	Baseline	Mid-season	Post-season	Baseline	Mid-season	Post-season	Baseline	Mid-season	Post-season	Baseline	Mid-season	Post-season
[7] positive response	8	8	4	30	8	4	n/a	29	19	64	25	15
[6]	15	15	12	48	33	25	n/a	32	24	33	29	13
[5]	22	13	13	21	24	20	n/a	11	12	9	8	13
[4] neutral response	37	26	22	10	7	9	n/a	4	3	7	7	14
[3]	22	10	6	4	3	1	n/a	0	1	2	5	3
[2]	9	3	2	3	1	0	n/a	1	1	0	2	1
[1] negative response	4	2	1	1	1	1	n/a	0	0	2	1	1

Supplementary Figure 2. Presentation of player responses in the constructs in the HAPA model motivational phase across one season in the Adductor group.

n = 117 at baseline, n = 77 at mid-season and n = 60 at post-reason. Note: Action self-efficacy was only rated at mid- and post-season. For constructs where players responded to more than one question, the averaged aggregated responses are shown in the figure. Abbreviations: HAPA — Health Action Process Approach, n/a — not applicable.

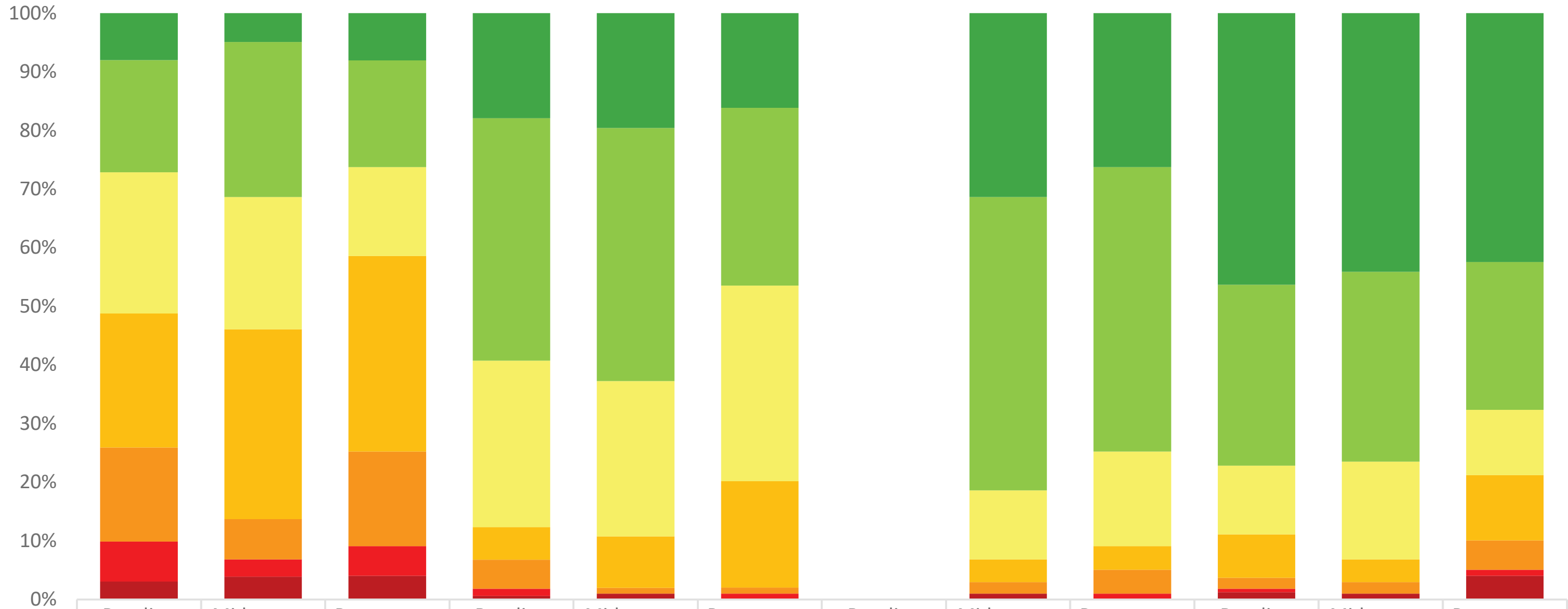

	Injury risk perceptions			Outcome expectancies			Action self-efficacy			Intention		
	Baseline	Mid-season	Post-season	Baseline	Mid-season	Post-season	Baseline	Mid-season	Post-season	Baseline	Mid-season	Post-season
[7] positive response	13	5	8	29	20	16	n/a	32	26	75	45	42
[6]	31	27	18	67	44	30	n/a	51	48	50	33	25
[5]	39	23	15	46	27	33	n/a	12	16	19	17	11
[4] neutral response	37	33	33	9	9	18	n/a	4	4	12	4	11
[3]	26	7	16	8	1	1	n/a	2	4	3	2	5
[2]	11	3	5	2	0	1	n/a	0	1	1	0	1
[1] negative response	5	4	4	1	1	0	n/a	1	0	2	1	4

Supplementary Figure 3. Presentation of player responses in the constructs in the HAPA model motivational phase across one season in the Comparison group.

n = 162 at baseline, n = 102 at mid-season and n = 99 at post-reason. Note: Action self-efficacy was only rated at mid- and post-season. For constructs where players responded to more than one question, the averaged aggregated responses are shown in the figure. Abbreviations: HAPA — Health Action Process Approach, n/a — not applicable.

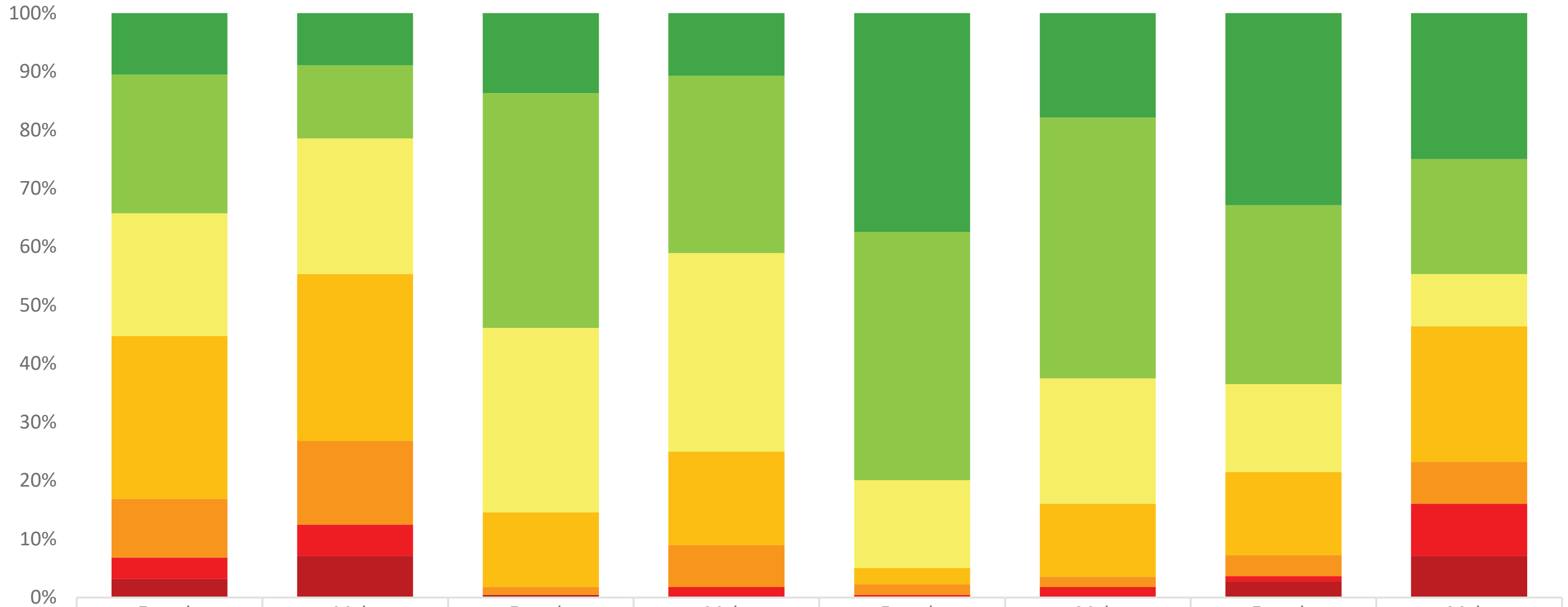

	Female	Male	Female	Male	Female	Male	Female	Male
	Injury risk perceptions		Outcome expectancies		Action self-efficacy		Intention	
[7] positive response	23	5	30	6	82	10	72	14
[6]	52	7	88	17	93	25	67	11
[5]	46	13	69	19	33	12	33	5
[4] neutral response	61	16	28	9	6	7	31	13
[3]	22	8	3	4	4	1	8	4
[2]	8	3	0	1	1	1	2	5
[1] negative response	7	4	1	0	0	0	6	4

Supplementary Figure 4. Presentation of player responses in the constructs in the HAPA model motivational phase at post-season, stratified by player sex.

n = 56 male players and n = 219 female players. Note: For constructs where players responded to more than one question, the averaged aggregated responses are shown in the figure. Abbreviations: HAPA — Health Action Process Approach.

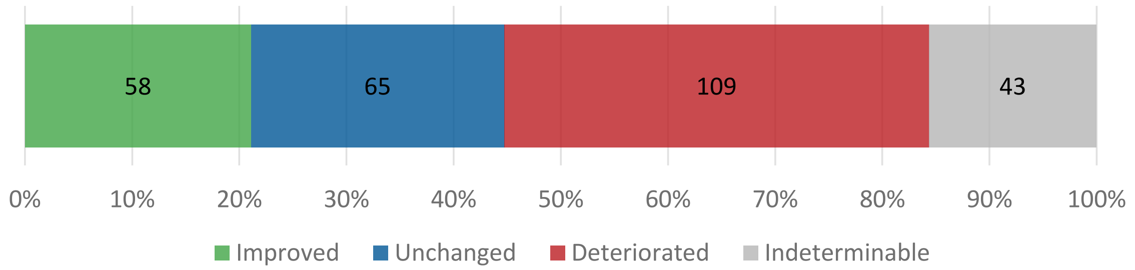

Supplementary Figure 5. Results from the analysis applying Paretian principles for the player ratings in the HAPA model motivational phase from baseline to post-season.

Note: Results illustrate the number of players whose ratings improved, were unchanged, deteriorated, or were indeterminable (where deteriorations in some constructs and improvements in the same number of constructs were seen) from baseline to follow-up. Likert responses 1–2 were considered negative, 3–5 neutral and 6–7 positive. Abbreviations: HAPA — Health Action Process Approach.

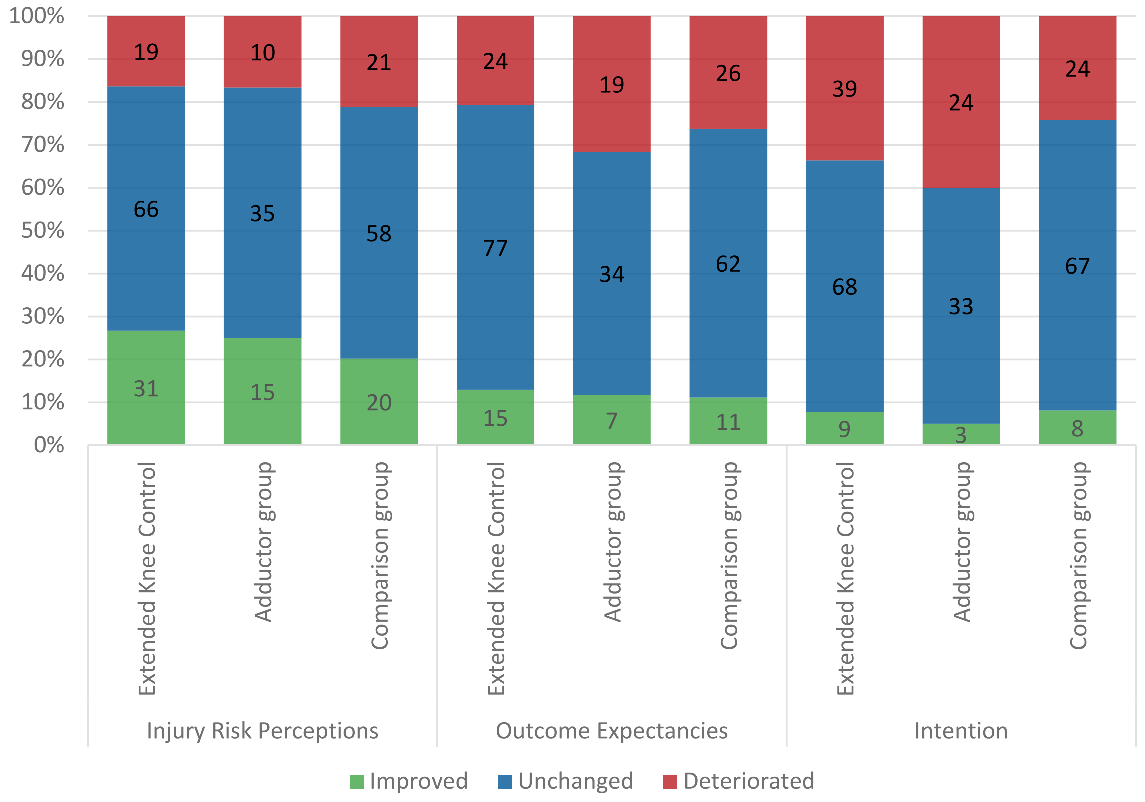

Supplementary Figure 6. Change in the ratings in injury risk perceptions, outcome expectancies, and intention for players in the respective groups from baseline to post-season.

Note: Results illustrate the number of players whose ratings improved, were unchanged, or deteriorated from baseline to follow-up. Likert responses 1–2 were considered negative, 3–5 neutral, and 6–7 positive.

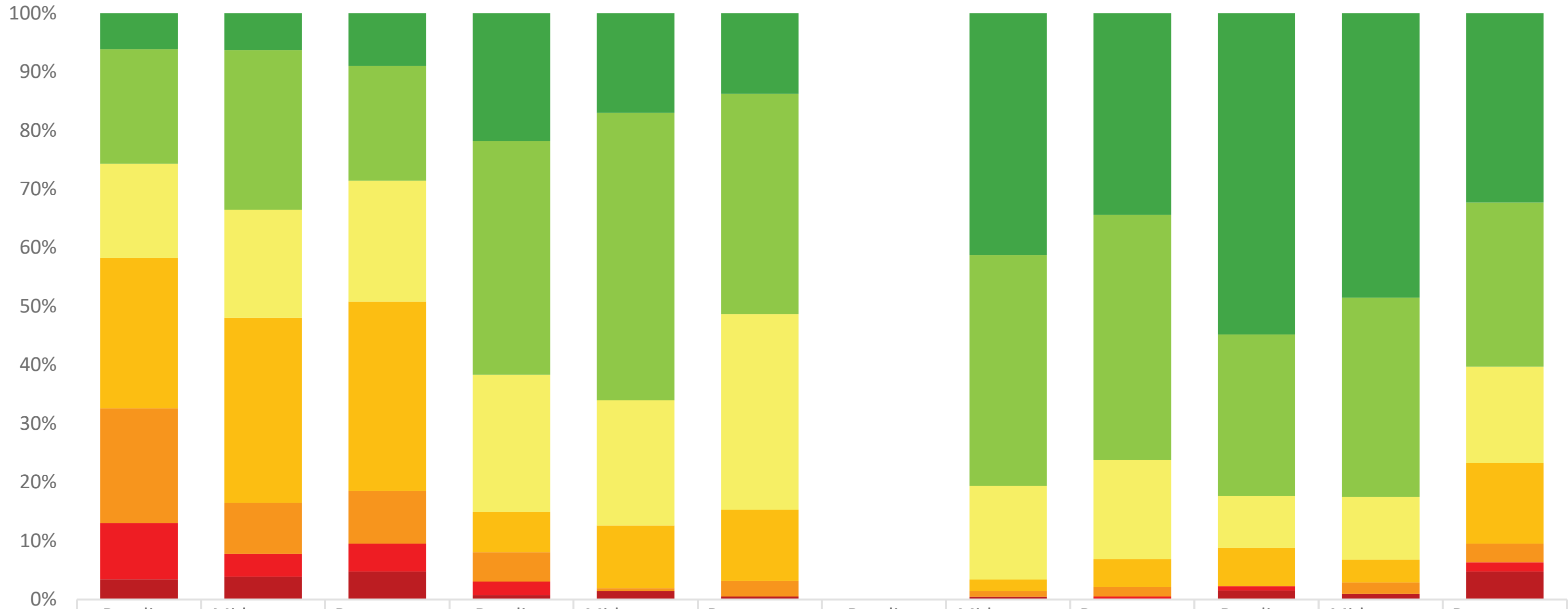

	Injury risk perceptions			Outcome expectancies			Action self-efficacy			Intention		
	Baseline	Mid-season	Post-season	Baseline	Mid-season	Post-season	Baseline	Mid-season	Post-season	Baseline	Mid-season	Post-season
[7] positive response	16	13	17	57	35	26	n/a	85	65	143	100	61
[6]	51	56	37	104	101	71	n/a	81	79	72	70	53
[5]	42	38	39	61	44	63	n/a	33	32	23	22	31
[4] neutral response	67	65	61	18	22	23	n/a	4	9	17	8	26
[3]	51	18	17	13	1	5	n/a	2	3	0	4	6
[2]	25	8	9	6	0	0	n/a	0	1	2	0	3
[1] negative response	9	8	9	2	3	1	n/a	1	0	4	2	9

Supplementary Figure 7. Ratings in the constructs in the HAPA model across one season for players who did not incur an injury during the season.

n = 261 responses at baseline, n = 206 at mid-season, and n = 189 at post-season. Note: For constructs where players responded to more than one question, the averaged aggregated responses are shown in the figure. Abbreviations: HAPA — Health Action Process Approach, n/a — not applicable.

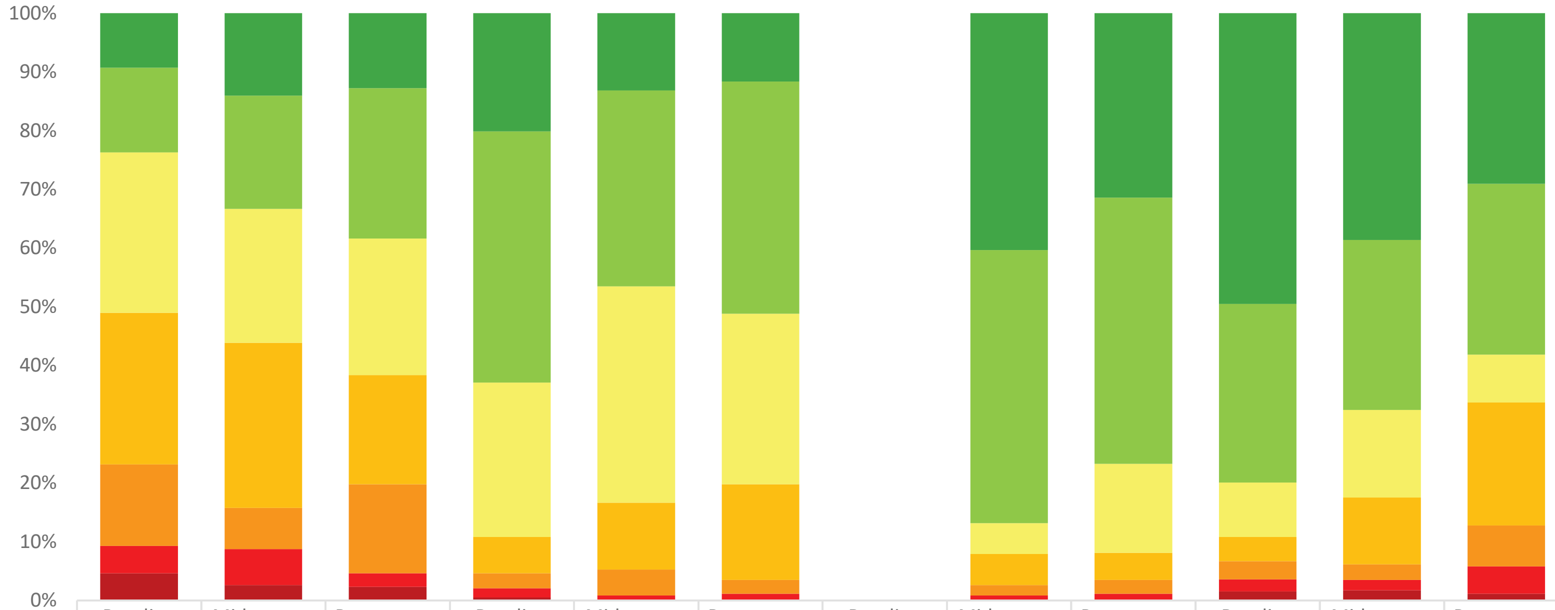

	Injury risk perceptions			Outcome expectancies			Action self-efficacy			Intention		
	Baseline	Mid-season	Post-season	Baseline	Mid-season	Post-season	Baseline	Mid-season	Post-season	Baseline	Mid-season	Post-season
[7] positive response	18	16	11	39	15	10	n/a	46	27	96	44	25
[6]	28	22	22	83	38	34	n/a	53	39	59	33	25
[5]	53	26	20	51	42	25	n/a	6	13	18	17	7
[4] neutral response	50	32	16	12	13	14	n/a	6	4	8	13	18
[3]	27	8	13	5	5	2	n/a	2	2	6	3	6
[2]	9	7	2	3	1	1	n/a	1	1	4	2	4
[1] negative response	9	3	2	1	0	0	n/a	0	0	3	2	1

Supplementary Figure 8. Ratings in the constructs in the HAPA model across one season for players who incurred an injury during the season.

n = 194 responses at baseline, n = 114 at mid-season, and n = 86 at post-season. Note: For constructs where players responded to more than one question, the averaged aggregated responses are shown in the figure. Abbreviations: HAPA — Health Action Process Approach, n/a — not applicable.

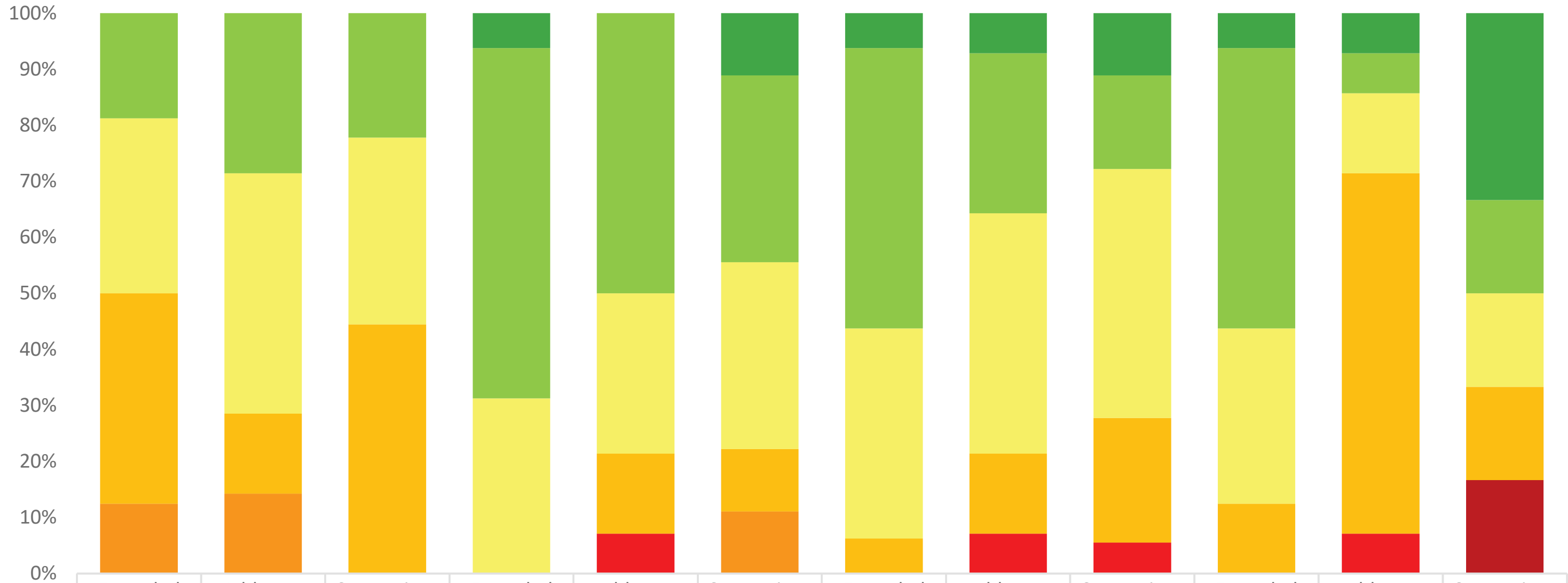

	Injury risk perceptions			Outcome expectancies			Action self-efficacy			Intention		
	Extended Knee Control	Adductor group	Comparison group	Extended Knee Control	Adductor group	Comparison group	Extended Knee Control	Adductor group	Comparison group	Extended Knee Control	Adductor group	Comparison group
[7] positive response	0	0	0	1	0	2	1	1	2	1	1	6
[6]	3	4	4	10	7	6	8	4	3	8	1	3
[5]	5	6	6	5	4	6	6	6	8	5	2	3
[4] neutral response	6	2	8	0	2	2	1	2	4	2	9	3
[3]	2	2	0	0	0	2	0	0	0	0	0	0
[2]	0	0	0	0	1	0	0	1	1	0	1	0
[1] negative response	0	0	0	0	0	0	0	0	0	0	0	3

Supplementary Figure 9. Presentation of coach responses in the constructs in the HAPA model motivational phase at post-season separated by intervention group.

n = 16 coaches in extended Knee Control, n = 14 coaches in the adductor group, and n = 18 coaches in the comparison group. Note: For constructs where coaches responded to more than one question, the averaged aggregated responses are shown in the figure. Abbreviations: HAPA — Health Action Process Approach.

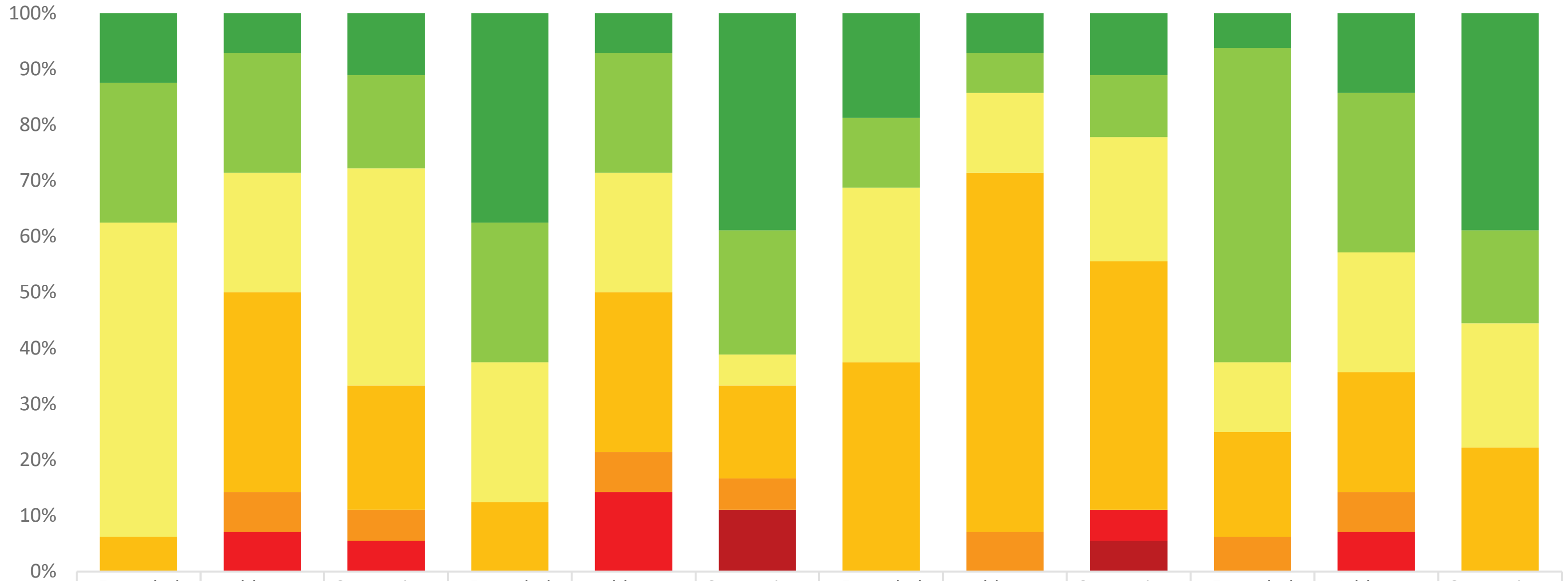

	Action planning			Maintenance self-efficacy			Coping planning			Recovery self-efficacy		
	Extended Knee Control	Adductor group	Comparison group	Extended Knee Control	Adductor group	Comparison group	Extended Knee Control	Adductor group	Comparison group	Extended Knee Control	Adductor group	Comparison group
[7] positive response	2	1	2	6	1	7	3	1	2	1	2	7
[6]	4	3	3	4	3	4	2	1	2	9	4	3
[5]	9	3	7	4	3	1	5	2	4	2	3	4
[4] neutral response	1	5	4	2	4	3	6	9	8	3	3	4
[3]	0	1	1	0	1	1	0	1	0	1	1	0
[2]	0	1	1	0	2	0	0	0	1	0	1	0
[1] negative response	0	0	0	0	0	2	0	0	1	0	0	0

Supplementary Figure 10. Presentation of coach responses in the constructs in the HAPA model goal-pursuit phase at post-season, separated by intervention group.

n = 16 coaches in extended Knee Control, n = 14 coaches in the adductor group, and n = 18 coaches in the comparison group. Note: For constructs where coaches responded to more than one question, the averaged aggregated responses are shown in the figure. Abbreviations: HAPA — Health Action Process Approach.

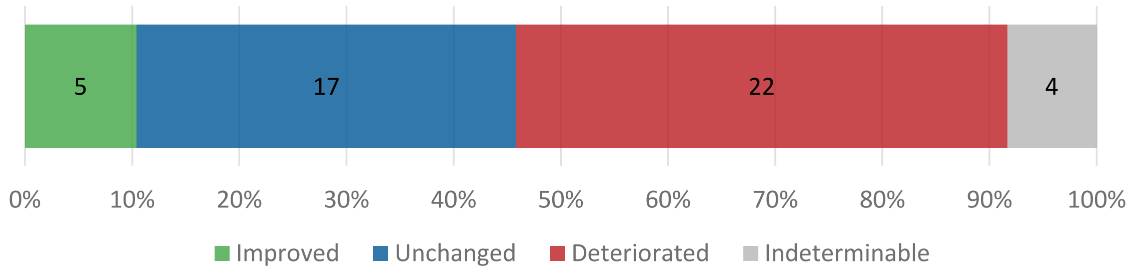

Supplementary Figure 11. Results from the analysis applying Paretian principles for the coach ratings in the HAPA model motivational phase from baseline to post-season.

Note: Results illustrate the number of coaches whose ratings improved, were unchanged, deteriorated, or were indeterminable (where deteriorations in some constructs and improvements in the same number of constructs were seen) from baseline to follow-up. Likert responses 1–2 were considered negative, 3–5 neutral, and 6–7 positive. Abbreviations: HAPA — Health Action Process Approach.

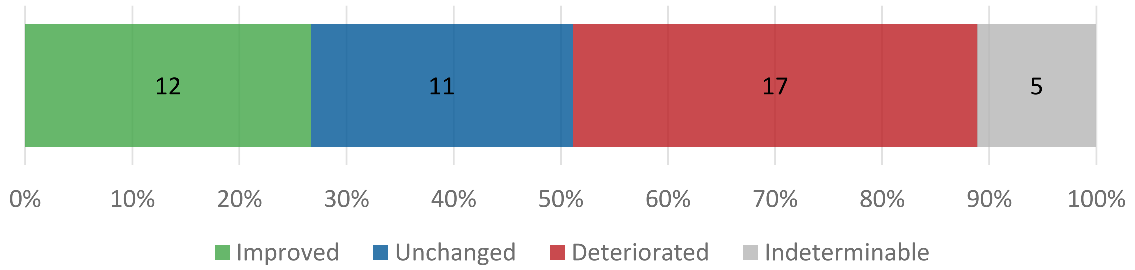

Supplementary Figure 12. Results from the analysis applying Paretian principles for the coach ratings in the HAPA model goal-pursuit phase from mid-season to post-season.

Note: Results illustrate the number of coaches whose ratings improved, were unchanged, deteriorated, or were indeterminable (where deteriorations in some constructs and improvements in the same number of constructs were seen) from baseline to follow-up. Likert responses 1–2 were considered negative, 3–5 neutral, and 6–7 positive. Abbreviations: HAPA — Health Action Process Approach.

Supplementary tables

Supplementary Table 1. Player ratings in the HAPA model motivational phase across one season.

		Baseline (n=455)	Mid-season (n=320)	Post-season (n=275)
**Injury risk perceptions**				
I expect I will sustain an injury sometime during this (next) season…	Median (IQR)	4.0 (2.0)	5.0 (2.0)	5.0 (2.0)
(1 likely –7 unlikely)	Likert score 1	18 (4.0)	11 (3.4)	11 (4.0)
	2	34 (7.5)	15 (4.7)	11 (4.0)
	3	78 (17.1)	26 (8.1)	30 (10.9)
	4	117 (25.7)	97 (30.3)	77 (28.0)
	5	95 (20.9)	64 (20.0)	59 (21.5)
	6	79 (17.4)	78 (24.4)	59 (21.5)
	7	34 (7.5)	29 (9.1)	28 (10.2)
**Outcome expectancies**	**Median (IQR)**	**6.0 (1.0)**	**6.0 (1.0)**	**6.0 (1.0)**
I believe many football injuries can be prevented	Median (IQR)	6.0 (1.0)	6.0 (1.0)	6.0 (1.0)
(1 do not agree – 7 agree)	Likert score 1	7 (1.5)	5 (1.6)	2 (0.7)
	2	13 (2.9)	4 (1.3)	5 (1.8)
	3	18 (4.0)	7 (2.2)	10 (3.6)
	4	38 (8.4)	25 (7.8)	23 (8.4)
	5	126 (27.7)	76 (23.8)	75 (27.3)
	6	177 (38.9)	135 (42.2)	99 (36.0)
	7	76 (16.7)	68 (21.3)	61 (22.2)
I believe my risk of injury will decrease (has decreased) from doing injury prevention training	Median (IQR)	6.0 (1.0)	5.0 (2.0)	5.0 (2.0)
(1 do not agree – 7 agree)	Likert score 1	8 (1.8)	5 (1.6)	6 (2.2)
	2	15 (3.3)	9 (2.8)	9 (3.3)
	3	12 (2.6)	13 (4.1)	13 (4.7)
	4	50 (11.0)	77 (24.1)	71 (25.8)
	5	137 (30.1)	107 (33.4)	101 (36.7)
	6	150 (33.0)	76 (23.8)	54 (19.6)
	7	83 (18.2)	33 (10.3)	21 (7.6)

Supplementary Table 1. Continued

		Baseline	Mid-season	Post-season
**Intention**				
I intend to regularly take part in injury prevention training together with my team this (next) season	Median (IQR)	7.0 (1.0)	6.0 (1.0)	6.0 (3.0)
(1 do not agree – 7 agree)	Likert score 1	7 (1.5)	4 (1.3)	10 (3.6)
	2	6 (1.3)	2 (0.6)	7 (2.5)
	3	6 (1.3)	7 (2.2)	12 (4.4)
	4	25 (5.5)	21 (6.6)	44 (16.0)
	5	41 (9.0)	39 (12.2)	38 (13.8)
	6	131 (28.8)	103 (32.2)	78 (28.4)
	7	239 (52.5)	144 (45.0)	86 (31.3)
**Action self-efficacy**	**Median (IQR)**	**n/a**	**6.0 (1.0)**	**6.0 (1.0)**
I have listened to my coach's instructions on how to do the preventive exercises	Median (IQR)	n/a	7.0 (1.0)	7.0 (1.0)
(1 do not agree – 7 agree)	Likert score 1		4 (1.3)	3 (1.1)
	2		1 (0.3)	2 (0.7)
	3		2 (0.6)	1 (0.4)
	4		5 (1.6)	15 (5.5)
	5		22 (6.9)	22 (8.0)
	6		101 (31.6)	88 (32.0)
	7		185 (57.8)	144 (52.4)
I have been able to do the preventive exercises correctly	Median (IQR)	n/a	6.0 (1.0)	6.0 (2.0)
(1 not confident – 7 confident)	Likert score 1		3 (0.9)	0 (0.0)
	2		2 (0.6)	4 (1.5)
	3		3 (0.9)	7 (2.5)
	4		15 (4.7)	16 (5.8)
	5		48 (15.0)	42 (15.3)
	6		112 (35.0)	93 (33.8)
	7		137 (42.8)	113 (41.1)
I have made 100% effort when we performed the preventive exercises	Median (IQR)	n/a	6.0 (2.0)	6.0 (2.0)
(1 do not agree – 7 agree)	Likert score 1		4 (1.3)	5 (1.8)
	2		1 (0.3)	2 (0.7)
	3		7 (2.2)	4 (1.5)
	4		13 (4.1)	23 (8.4)
	5		67 (20.9)	72 (26.2)
	6		122 (38.1)	98 (35.6)
	7		106 (33.1)	71 (25.8)

Supplementary Table 1. Continued

		Baseline	Mid-season	Post-season
**Additional question (not related to specific HAPA construct)**				
If my team uses preventive exercises the rest of the (next) season, I think it is…	Median (IQR)	n/a	6.0 (2.0)	6.0 (2.0)
(1 bad – 7 good)	Likert score 1		1 (0.3)	6 (2.2)
	2		1 (0.3)	9 (3.3)
	3		4 (1.3)	6 (2.2)
	4		37 (11.6)	36 (13.1)
	5		51 (15.9)	56 (20.4)
	6		120 (37.5)	71 (25.8)
	7		106 (33.1)	91 (33.1)

Values are median (interquartile range) or n (%) for the Likert scores. Each question is presented with the anchors (1 and 7) on the Likert scale. Each construct in the HAPA model is given in bold in the left column, and aggregated scores for each construct is presented in bold for constructs incorporating more than one question. Abbreviations: HAPA–Health Action Process Approach, IQR–interquartile range, n/a — not applicable.

Supplementary Table 2. Player ratings in the HAPA model motivational phase separated by intervention group.

	Extended Knee Control			Adductor programme			Comparison group		
	Baseline (n=176)	Mid-season (n=141)	Post-season (n=116)	Baseline (n=117)	Mid-season (n=77)	Post-season (n=60)	Baseline (n=162)	Mid-season (n=102)	Post-season (n=99)
**Injury risk perceptions**									
I expect I will sustain an injury sometime during this (next) season… (likely – unlikely)	4.0 (3.0)	5.0 (2.0)	5.0 (2.0)	4.0 (2.0)	4.0 (2.0)	4.0 (2.0)	5.0 (3.0)	5.0 (2.0)	4.0 (3.0)
**Outcome expectancies**	**6.0 (1.0)**	**6.0 (1.0)**	**6.0 (1.0)**	**6.0 (2.0)**	**6.0 (1.0)**	**5.0 (1.0)**	**6.0 (1.0)**	**6.0 (1.0)**	**5.0 (1.0)**
I believe many football injuries can be prevented (do not agree – agree)	6.0 (1.0)	6.0 (1.0)	6.0 (1.0)	6.0 (1.0)	6.0 (2.0)	6.0 (1.75)	6.0 (1.0)	6.0 (1.0)	6.0 (1.0)
I believe my risk of injury will decrease (has decreased) from doing injury prevention training (do not agree – agree)	6.0 (1.0)	5.0 (2.0)	5.0 (2.0)	6.0 (1.0)	5.0 (1.5)	5.0 (1.0)	5.5 (1.0)	5.0 (1.25)	5.0 (2.0)
**Intention**									
I intend to regularly take part in injury prevention training together with my team this (next) season (do not agree – agree)	7.0 (1.0)	7.0 (1.0)	6.0 (2.75)	7.0 (1.0)	6.0 (2.0)	5.0 (2.75)	6.0 (1.0)	6.0 (2.0)	6.0 (2.0)
**Action self-efficacy**	**n/a**	**6.0 (1.0)**	**6.0 (1.0)**	**n/a**	**6.0 (1.0)**	**6.0 (2.0)**	**n/a**	**6.0 (1.0)**	**6.0 (2.0)**
I have listened to my coach's instructions on how to do the preventive exercises (do not agree – agree)	n/a	7.0 (1.0)	7.0 (1.0)	n/a	7.0 (1.0)	7.0 (1.0)	n/a	6.0 (1.0)	6.0 (1.0)
I have been able to do the preventive exercises correctly (not confident – confident)	n/a	7.0 (1.0)	6.0 (1.0)	n/a	6.0 (2.0)	6.0 (2.0)	n/a	6.0 (2.0)	6.0 (2.0)
I have made 100% effort when we performed the preventive exercises (do not agree – agree)	n/a	6.0 (1.0)	6.0 (2.0)	n/a	6.0 (2.0)	6.0 (1.75)	n/a	6.0 (2.0)	6.0 (1.0)
**Additional question (not related to specific HAPA construct)**									
If my team uses preventive exercises the rest of the (next) season, I think it is… (bad – good)	n/a	6.0 (2.0)	6.0 (2.75)	n/a	6.0 (2.0)	5.0 (2.0)	n/a	6.0 (1.0)	7.0 (1.0)

Values are median (interquartile range). Each question is presented with the anchors (1 and 7) on the Likert scale. Each construct in the HAPA model is given in bold in the left column, and aggregated scores for each construct is presented in bold for constructs incorporating more than one question. Abbreviations: HAPA—Health Action Process Approach, n/a — not applicable.

Supplementary Table 3. Coach ratings in the HAPA model motivational and goal-pursuit phases.

	Baseline (n=59)	Mid-season (n=49)	Post-season (n=48)
**MOTIVATIONAL PHASE**			
**Injury risk perceptions**	**5.0 (1.0)**	**5.0 (1.5)**	**5.0 (1.0)**
What do you think about the overall injury risk in football? (low–high)	5.0 (1.0)	5.0 (0.0)	5.0 (0.75)
What do you think about the injury risk in the team that you coach? (low–high)	4.0 (2.0)	5.0 (1.0)	4.0 (2.0)
**Outcome expectancies**	**6.0 (0.0)**	**6.0 (1.0)**	**6.0 (1.0)**
I believe many injuries can be prevented in football (do not agree–agree)	6.0 (1.0)	6.0 (1.0)	6.0 (1.0)
I believe the injury risk in my team will decrease (has decreased) from regular injury prevention training… (do not agree–agree)	6.0 (1.0)	5.0 (1.0)	5.0 (1.0)
**Action self-efficacy**	**5.0 (2.0)**	**5.0 (1.5)**	**5.0 (1.0)**
My knowledge about preventing injuries in football is… (inadequate–adequate)	5.0 (1.0)	5.0 (1.0)	5.0 (0.0)
My practical ability to lead specific injury prevention training in my team is… (inadequate–adequate)	5.0 (2.0)	5.0 (2.0)	5.0 (1.75)
**Intention**			
I intend to prioritise injury prevention training in my team this (next) season… (do not agree – agree)	6.0 (1.0)	6.0 (1.0)	5.0 (2.0)
**GOAL-PURSUIT PHASE**			
**Action planning**			
I have concrete plans for how to instruct the players in injury prevention exercises… (do not agree – agree).	5.0 (1.0)	5.0 (2.0)	5.0 (2.0)
**Maintenance self-efficacy**			
I believe I will be able to continue using injury prevention exercises in my team this (next) season… (do not agree – agree)	n/a	6.0 (2.0)	6.0 (3.0)
**Coping planning**			
I have plans for how to work around barriers for continued injury prevention programme use (do not agree – agree)	n/a	4.0 (1.0)	4.0 (1.0)
**Recovery self-efficacy**			
If my team stops using injury prevention training, I am certain that we can start using it again (do not agree – agree)	n/a	6.0 (1.0)	6.0 (2.0)

Values are median (interquartile range). Each question is presented with the anchors (1 and 7) on the Likert scale. Each construct in the HAPA model is given in bold in the left column, and aggregated scores for each construct is presented in bold for constructs incorporating more than one question. The two HAPA phases are presented in capital letters. Abbreviations: HAPA — Health Action Process Approach, n/a — not applicable.
